# Correspondence Respecting the Quarantine Laws. Presented to Parliament

**Published:** 1846-10-01

**Authors:** 


### Correspondence respecting the Quarantine Laws, &c. Presented to Parliament. Folio, pp. 48. 1846.

We have alluded in a former page to the negociations that have of late years been carried on in France, as well as in our own country, upon this most important subject. The following are the particulars.

In 1838, a proposal was made by the French to the British government to promote the formation of a congress of delegates from the various European states having ports in the Mediterranean, for the purpose of agreeing upon some uniform system of Quarantine regulations to be adopted by and binding upon all. The British government at once acceded to the proposal. Austria also, which had been applied to by France about the same time, intimated her assent to its general principle and substance, only with some modification in the details. Difficulties, however, were subsequently started by the Austrian government, and the matter dropped entirely until the year 1843; when Lord Aberdeen again took it into consideration, and invited the French and Austrian governments to join with him in carrying out the proposal made by the former in 1838. France expressed her concurrence; but Austria considered that the establishment of any conference or congress, as proposed, would be premature until exact information was procured from competent medical men upon the following three points:

1. The minimum and maximum of the terms of quarantine to be fixed for persons.
2. The terms of quarantine necessary for goods and merchandize.
3. The best measures to be adopted for the disinfection of objects that are susceptible of contagion.

Prince Metternich intimated, at the same time, that a period of six months would probably be required to obtain this preparatory information, before the proposed congress of delegates could proceed satisfactorily to the determination of the various questions to be submitted to them.

We have already seen what steps the French government took to procure the most accurate instructions on the several points to which the prince referred.

The British government also, anxious that all just or alleged causes of delay might be removed, dispatched about the same time, viz: in October 1844, Sir William Pym, the Superintendant of Quarantine in this country, to visit all the ports in the Mediterranean where quarantine establishments and lazarettos existed, and to draw up a report upon them.

After describing the various places which he visited, and briefly mentioning the quarantine regulations that exist in each, Sir William makes the general remark that " the periods of Quarantine both as to the persons and merchandize may be very considerably reduced, particularly with reference to vessels arriving from places with clean bills of health, and in some instances altogether abolished, such as upon vessels from the Black Sea and those crossing the Atlantic; and that many of the unnecessary, vexatious, and expensive regulations, more particularly in the Italian States, may be discontinued."*

As a matter of course, Sir William takes it for granted that the plague is contagious (communicable by contact, we presume), and that the contagion may be transmitted not only by the sick themselves but by various *fomites;* for we read that " it will be necessary to decide upon a list of articles of merchandize that are supposed to require purification, under the impression that they have been contaminated by persons having the plague, and the period of time required for their purification, together with the best means of doing so. To effect this, it may be necessary to examine practical men from different lazaret establishments, the superintendants of quarantine, captains of lazarets, and the medical men attached to these establishments." Some of these parties here named are not, we fear, the most likely to come to very sober decisions upon the points under consideration; they are contagionists *par metier*.

The date of Sir Wm's letter, from which these extracts are taken, is June 1845. His next letter, dated September of the same year, is occupied with answers to the three questions or points of enquiry which Prince Metternich considered to require solution, before any step could be wisely taken to establish a congress. To the *first* on the list—as to the minimum and maximum of the terms of quarantine necessary for persons—he gives the following reply:

" It appears to me that this question, and the one of greatest importance in this enquiry (the incubation of plague in the human system), was decided by the almost unanimous opinion of eighteen medical men in the Levant, by their replies to queries as published in the Parliamentary Papers; all of them, however much they differed in opinion upon the subject of contagion, agreed upon the short

* The health authorities of Palermo acknowledged to Sir William that they had run bars of iron through the fire, and had washed sugar-hogsheads with lime, for the purpose of destroying contagion! Very lately, a vessel from England was put under quarantine at Messina, because a report had appeared in the newspapers of a fever having prevailed at Glasgow!!

period of incubation, viz. from three to ten days, with one exception, Dr. Floquin, of Smyrna, who put down fifteen days as the maximum; and the opinion of those medical men is confirmed by the inclosed valuable document. No. 1, being a return of 5240 individuals who had undergone the *spoglio* in the lazaret of Alexandria in the course of four years, out of which number forty-three were attacked with plague, and all of them before the eighth day after the operation of *spoglio*."

The answer to the *second* question contains an admission that is so truly important, in a commercial as well as in a medical point of view, that we request our reader's special attention to it:

"It is difficult to obtain any decided evidence upon this question, as during my Quarantine mission *I could not ascertain that any one case of plague had been produced in any one of the various lazarets that I visited, in consequence of the manipulation of merchandize* (the Italics are ours.—*Rev.*); and as in the principal lazarets in the Mediterranean (Marseilles and Malta) they have gradually abolished the *serenos* (probationary airings), and reduced the period of depuration of goods with foul bills to twenty-one days, it does not appear that this period could be further reduced with such cargoes as cotton, if exposure to the atmosphere is to be considered necessary; as it will take the full time, according to the present practice of opening first one side of the bale for a certain period, then making up that side and opening the other for the same time, making in fact the quarantine upon cotton only ten days, which last period will be sufficient for small cargoes or parcels of merchandize, which can be at once opened and exposed to the air."

On the *third* point—as to the best means of disinfecting objects susceptible of contagion—Sir W. very naively remarks; "from what I have stated as to the non-appearance of plague in any one instance from the manipulation of merchandize in Lazarets, the present practice of exposing goods to the atmosphere for a certain period, appears to have been attended with success." He seems never to have even so much as dreamed of the possibility that the goods possessed no contagious property whatsoever; and yet, strange to say, he was aware, all the while, that not a single instance could be produced of the plague having been ever communicated by the manipulation of merchandize, in the lazarets which he visited! Such are the blinding effects of old deep-rooted prepossessions on the mental vision even of the most experienced observers.

The following brief notice of the vessels which have arrived at Malta with the plague on board, and have been duly depurated in the lazaretto there, since the island has been in possession of Great Britain (not including the plague of 1813), will be found to contain some interesting particulars, serving to illustrate and confirm several of the most important positions in the French Report. The details will not be deemed tedious or unnecessary by any who wish to understand thoroughly the important subject under consideration.

1819.—A Maltese vessel, laden with oil and soap, arrived on the 27th of March from Susa, from which she had sailed on the 20th with a foul bill, in consequence of the plague prevailing there; from 15 to 18 persons were dying daily. The day before sailing, one of the crew had been taken with symptoms of fever. On the 21st, vomiting with delirium set in, and next day he died. The master stated that there were no external marks of plague during life, but that several petechiæ were observed on the belly and thighs after death. Four of the remaining five persons of the crew were attacked with the plague, while in the lazaretto; one on the 3rd, and three on the 4th of April. Of these four cases, two proved fatal. *The health guard, and two persons* (all from pratique) *who nursed the sick, were not attacked.*

1821.—A Maltese brig, laden with beans, arrived on the 21st of March from Alexandria, with a foul bill, having 14 of a crew and 8 passengers on board; two of the passengers and one of the crew had died during the voyage. On the 27th February, the day before sailing from Alexandria, ten passengers were received on board. Of these, eight were in good health; but two, a woman and her daughter, were sickly, having been suffering from diarrhœa. Both died, the mother on the 2nd March, and the daughter on the 16th; no marks of plague were observed, it was said, on examination of the bodies. Besides these cases, one of the crew, who had suffered much from sea-sickness and had taken no food, became feverish and delirious on the 17th; then profuse diarrhœa came on, and he died about midnight. Several petechiæ were observed upon the body. While in the lazaret, no fewer than ten of the crew and four of the passengers were taken ill; of these 14 cases, 12 proved fatal.

On the 28th of March, a health-guard and four sailors were embarked from pratique on board the infected vessel, to depurate it and land the cargo. One of the sailors was attacked on the 2nd of April and recovered; he had had the plague in Malta in 1813. Another sailor was attacked; he also recovered.

*No mention is made of any of the official inmates of the lazaretto or attendants upon the sick having suffered.*

1828.—On the 13th of June 1828, the Russian frigate "Castor," with 281 of a crew, arrived at Malta from Armiro, from which she had sailed on the 21st of May. She had previously left Malta in pratique on the 30th of April for Navarino and Modone. On the 3rd of May, she was in company with three other Russian ships-of-war; they had captured a Turkish corvette, which had sailed the day before from Modone for Alexandria, having on board 600 individuals, including the crew, invalids, sick, and wounded from Ibrahim Pacha's army. (It is not to be wondered at that some malignant fever prevailed amid such an assemblage). The captured corvette was manned by 15 sailors from the Castor, and men from the other Russian ships; at the same time, 200 out of the 600 of the corvette's company were taken on board the Castor, and, on the 11th of May, were all landed on the coast of Morea; the Castor receiving back the 15 of her crew from the corvette. On the 17th, she arrived at Armiro; and on that day a sailor was seized with headache, vomiting, and delirium. Next day, another was taken ill with similar symptoms. On the 19th the former patient died, the body exhibiting externally nothing indicating plague. On the 20th, the latter was carried off; and still no marks of plague were visible on the body. On the same day, a third sailor was similarly attacked; he died on the 24th, but without having exhibited any of the outward signs of plague. On the 2nd of June, while at sea, a fourth sailor was taken ill; but, besides the general symptoms observed in the three preceding cases, this patient had a swelling under the right arm; he died on the 9th. As the surgeon now declared the disease to be a contagious pestilential fever, the frigate was immediately ordered to Malta, there to undergo quarantine and depuration. *No new cases occurred either on board or at the lazaret during the Castor's stay.*

1835.—A Russian brig, with a cargo of bales of cotton and linen and other susceptible goods, arrived from Alexandria on the 2nd May with a foul bill, bound for Leghorn, and having 13 persons (originally 15) on board. On the 11th of April, whilst at Alexandria, one of the sailors fell overboard; on the same day, he was taken ill with a pain in the chest. He, however, got better, and continued so until the day after leaving Alexandria (this was on the 18th), when he became worse, and on the following day he died: no marks of plague were observed on the body. On the 27th, whilst off Candia, another sailor was taken ill with pain in the chest, extreme debility and spitting of blood; he continued daily getting worse until the 28th, when he died. Several blue spots were observed on the body. On the 1st of May, being near Girgenti, a third sailor was seized with violent headache and general debility; these symptoms continued until the following day, when he died. On the same day (2nd), a fourth sickened in a similar manner to the others. The rest of the crew remained in health. During the stay in the lazaretto, five other cases occurred, and four of them proved fatal. In these four cases, there were petechiæ and carbuncles: in the fifth there were not any. *Again, there is no mention of any of the attendants in the lazaretto having been affected.*

1837.—An Ottoman vessel arrived on the 22nd February from Tripoli (which she had left on the 15th) with a foul bill of health, having a crew of 6 persons and 52 passengers on board. One of the latter had been taken ill on the day of leaving Tripoli, and continued so all the voyage: he had been kept apart in a small boat on deck. When taken to the lazaretto, two bubos were visible in the groins: the man recovered. *This is all that is stated respecting this vessel: the disease, therefore, did not spread to any one.*

1837.—An Ottoman vessel, "laden with susceptible goods and beans," which had sailed from Tripoli with 11 of a crew and 22 passengers on the 10th of February, arrived at Malta on the 23rd. One of the passengers fell overboard during the passage, and was drowned. Two of the passengers and one of the crew sickened in the lazaretto—one on the 27th, another on the 28th, and the third on the 8th of March; they all died on the second day after the attack. In one case there was a bubo, in another there were petechiæ.

Another vessel arrived about the same time from Tripoli with a foul bill, and two persons sick. On being conveyed to the lazaretto, symptoms of plague appeared in both. One died. "*Two of the crew, who attended and nursed their fellow-sailor in free communication with him, continued in perfect health.*

1840.—H. M. steamer "Acheron" from Alexandria arrived at Malta 27th April 1840, with a foul bill of health in seven days, with eighteen passengers and forty-eight persons in crew, having brought the mails, several parcels, letters, and two horses, all well on board. On the 29th, early in morning, the health-guard, who was put on board on the day of her arrival, reported to the captain of the lazaretto that one of the crew, a boy, during the night, at about 9 P.M., died, and that one of the stewards was seriously ill. The corpse and the sick steward having been landed in the lazaretto and examined by the physician, evident symptoms of plague were observed upon them. "*The persons, who attended and nursed the deceased, continued well.*"

1841.—On the 9th of March, H.M. frigate "Castor" arrived in 15 days from Kaiffa, having had 13 cases (two were doubtful) of plague on board; of which 9 had proved fatal. Four of the men had been taken ill on shore at Kaiffa, before the frigate sailed. On the 23d of February, four other men were sent from shore to be put on the sick list. In one or two of the cases, the symptoms were those of gastric fever; but in others the fever was truly malignant, and was accompanied with glandular swellings in the groins and axillæ. In one case the cervical, and in the other the sub-maxillary, glands were affected; in the latter instance, the patient was to all appearance recovering from the original disease, but was carried off by the diseased state of the tongue and fauces, the viscid secretions therefrom having produced sudden suffocation. In one case, the symptoms resembled those of *delirium tremens*, and were treated as such.

It is unnecessary to enter into farther particulars: suffice it to say that there is not a vestige of proof that the disease was communicated, in a single instance, from the sick either to the healthy on board or to any one in the lazaretto.

1841.—An Ottoman brig, in ballast, arrived from Alexandria on the 26th of May, having 15 persons in crew, and 180 passengers (haggis). She had sailed from Alexandria on the 8th. One of the passengers had died during the voyage; and three others had sickened, when in sight of the island. While in the lazaretto, 13 cases of plague took place, and 10 of them proved fatal. Of these, one occurred in a Maltese boatman, who was put in quarantine, from pratique, with one of the patients on the 28th. No other particular is mentioned.

A month afterwards, an Austrian brig, in ballast, arrived from Alexandria, which she had left with a foul bill of health, and having a crew of nine persons, and 105 passengers: she was bound for Tangiers. Eight of the passengers died on the voyage. While in the lazaretto, other three cases took place. Two of these proved fatal; the third, which occurred in a health-guard who had put in quarantine with the sick, did well.

The last instance of imported plague in the lazaretto of Malta was that of an Ottoman brig, which arrived in July of the same year, in 37 days from Alexandria, with a foul bill of health, 87 persons on board, and laden with linen, flax, beans, &c. No casualty occurred during the voyage. While in quarantine, four deaths took place; one from dysentery, one from inflammation of the brain, one from enteritis, and one from (what is called) pestilential bubo.

Now these various facts, drawn from the experience of the quarantine authorities at Malta during the last 25 years, appear to us to afford convincing testimony as to the very little risk of the transmissibility of the plague, away from an epidemic focus of the disease. Here we have an account—unexceptionably authentic, be it remembered—of between 40 and 50 cases of recognised plague having occurred in the lazaretto of Malta, without the disease having been communicated in a single instance to any of the *employés* in that establishment. The only cases which occurred among those in pratique were four; and what, pray, were the circumstances in which these persons were attacked? Two were of a party which had been put on board an infected vessel, to clean her out; and the remaining two were men who, from pratique, were put in quarantine and confined along with the sick—most probably in a small room or chamber.

The critical reader will have remarked how promptly and easily the disease was arrested, and how small the number and mortality of cases were on board the two ships of war, compared with the merchant vessels, crowded as these latter often were with poor filthy passengers, who had come direct from a focus of infection. Most of these vessels moreover, it will be observed, had foul bills of health upon their arrival.

So much for the results of infected vessels which have arrived at Malta within the last 25 years. Let us now see what has occurred at Marseilles during the same period. Our information upon this point is derived from the French Report, which has been already analysed at so great length; but we have deemed it better to introduce the details here, for more convenient comparison with those just given.

1819.—A Swedish vessel arrived at Marseilles on the 1st of May from Susa, which she had left on the 15th of April, and from Tunis, which she left on the 20th: both places were at these dates afflicted with epidemic plague. She had a crew of 12 persons; and, besides them, 17 passengers had been received on board at Tunis. On the 25th, one of the sailors was attacked with plague; he died. On the 26th, another of the sailors fell sick; but he recovered after a very tedious convalescence. Besides these deaths on board, a woman and her infant died: the latter of dentition, the former of what was supposed to be milk-fever. The rest of the crew and passengers remained healthy.

On the 12th of May, one of the health-guards, who had been put on board the vessel, was taken ill: he died on the 16th.

*Not a word is said of any other person, either in the vessel or in the lazaretto, suffering.*

1825.—A vessel, laden with cotton, hides, and wool, arrived on the 30th June from Alexandria, which she had left on the 29th of May. On the 5th of June, one man was taken ill and died on the 9th with symptoms of plague. A boy fell sick on the 16th, and died on the 19th, on which day the captain also was attacked: he died on the sixth day afterwards. Another man was taken ill within a few hours of the arrival of the brig at Marseilles; he was without delay conveyed to the infirmary of the lazaretto. The constitutional symptoms were by no means severe; two bubos had formed in the left groin, but neither of them suppurated. This man recovered. There was another case, in which the symptoms were comparatively very mild.

*No mention is made of any of the attendants in the lazaretto being infected.*

1837.—The "Leonidas," a post-office steamer, having on board 47 of crew and 18 passengers, arrived at Marseilles on the 9th of July, having left Constantinople on the 27th of June, Smyrna on the 30th, and Syra on the 1st of July. Passengers in quarantine had been embarked and disembarked both at Syra and at Leghorn; and at Malta, too, several passengers had been taken on board, but all in quarantine. Upon her arrival at Marseilles, one of the stokers was affected with low fever; he died next day. On examining the body on the 11th, it is stated that the brain and intestines were found to exhibit marks of violent inflammation; but that there was no appearance of bubos, carbuncles, or petechiæ. On the same day, another man, who was in the habit of sleeping in the same bed with the last patient, was visited by the lazaretto physician. He had been suffering for two days with severe headache, and within the last 24 hours, a painful swelling had made its appearance on the upper part of the left thigh. The case was regarded as very suspicious. The tumour became considerably larger, and the patient was in a state of great prostration with occasional delirium. On the 15th, petechiæ were observed on various parts of the body. On the 16th, a carbuncle appeared over the left ankle; the bubo had shrunk considerably; diarrhœa, vomiting, and delirium were the chief constitutional symptoms. The patient died next day. No *post-mortem* examination was made.

On the 20th, the cook of the Leonidas was taken suddenly ill in the lazaretto, with intense headache, vomiting, and great prostration. Besides these symptoms, there was a painful swelling at the lower part of the right groin. Next morning he was bled from the arm. On the 22nd, the headache was very severe, there was frequent vomiting of a greenish matter, the tongue was foul, and the patient was wandering in his ideas, and occasionally delirious. On the 23rd, the symptoms were more unfavourable; profuse diarrhœa had set in; the patient was in a state of collapse. Death took place on the 24th. When examined on the following day, the body was spotted with blueish patches in different places; the tumour in the groin had sunk down very much.

*No mention is made of any of the lazaretto attendants having suffered; not even Dr. Chevillon, who had most heroically waited upon the two last patients with the greatest assiduity.*

We shall make no comments on the numerous facts which have now been adduced;—facts too, be it remembered, drawn from the official records of lazarettos, the very places where we may be sure that the most would have been made of every event which seemed to favour in any measure the doctrine of infection, and the consequent necessity of strict Quarantines.

The amount of *materiel*, which we have submitted to the reader's attention in the present number of this Journal, will enable him—provided always he not only reads, but marks, learns, and inwardly digests its details—to form tolerably accurate conclusions on the important questions which its consideration involves. This is the more necessary at the present time, as the subject of quarantine enactments will be brought before Parliament in the course of next Session; and it therefore surely behoves all medical men to be acquainted with whatever has been made known by those who have had competent opportunities of observation. The present Government has pledged itself to the working out of several much-needed social reforms. We sincerely trust that the re-organization of our quarantine establishments may be one which meets with its earliest and most searching investigation.

